# Upregulation of the endothelin A (ET_A_) receptor and its association with neurodegeneration in a rodent model of glaucoma

**DOI:** 10.1186/s12868-017-0346-3

**Published:** 2017-03-01

**Authors:** Nolan R. McGrady, Alena Z. Minton, Dorota L. Stankowska, Shaoqing He, Hayden B. Jefferies, Raghu R. Krishnamoorthy

**Affiliations:** 1 North Texas Eye Research Institute, University of North Texas Health Science Center, 3500 Camp Bowie Blvd, Fort Worth, TX 76107 USA; 2 Texas College of Osteopathic Medicine, University of North Texas Health Science Center, 3500 Camp Bowie Blvd, Fort Worth, TX 76107 USA

**Keywords:** Primary open angle glaucoma (POAG), Intraocular pressure (IOP), Endothelin receptor A (ET_A_), Endothelin receptor B (ET_B_), Endothelin-1 (ET-1), Endothelin-3 (ET-3), Retinal ganglion cells (RGCs), Neurodegeneration

## Abstract

**Background:**

Primary open angle glaucoma is a heterogeneous group of optic neuropathies that results in optic nerve degeneration and a loss of retinal ganglion cells (RGCs) ultimately causing blindness if allowed to progress. Elevation of intraocular pressure (IOP) is the most attributable risk factor for developing glaucoma and lowering of IOP is currently the only available therapy. However, despite lowering IOP, neurodegenerative effects persist in some patients. Hence, it would be beneficial to develop approaches to promote neuroprotection of RGCs in addition to IOP lowering therapies. The endothelin system is a key target for intervention against glaucomatous neurodegeneration. The endothelin family of peptides and receptors, particularly endothelin-1 (ET-1) and endothelin B (ET_B_) receptor, has been shown to have neurodegenerative roles in glaucoma. The purpose of this study was to examine changes in endothelin A (ET_A_) receptor protein expression in the retinas of adult male Brown Norway rats following IOP elevation by the Morrison’s model of ocular hypertension and the impact of ET_A_ receptor overexpression on RGC viability in vitro.

**Results:**

IOP elevation was carried out in one eye of Brown Norway rats by injection of hypertonic saline through episcleral veins. After 2 weeks of IOP elevation, immunohistochemical analysis of retinal sections from rat eyes showed an increasing trend in immunostaining for ET_A_ receptors in multiple retinal layers including the inner plexiform layer, ganglion cell layer and outer plexiform layer. Following 4 weeks of IOP elevation, a significant increase in immunostaining for ET_A_ receptor expression was found in the retina, primarily in the inner plexiform layer and ganglion cells. A modest increase in staining for ET_A_ receptors was also found in the outer plexiform layer in the retina of rats with IOP elevation. Cell culture studies showed that overexpression of ET_A_ receptors in 661W cells as well as primary RGCs decreases cell viability, compared to empty vector transfected cells. Adeno-associated virus mediated overexpression of the ET_A_ receptor produced an increase in the ET_B_ receptor in primary RGCs.

**Conclusions:**

Elevated IOP results in an appreciable change in ET_A_ receptor expression in the retina. Overexpression of the ET_A_ receptor results in an overall decrease in cell viability, accompanied by an increase in ET_B_ receptor levels, suggesting the involvement of both ET_A_ and ET_B_ receptors in mediating cell death. These findings raise possibilities for the development of ET_A_/ET_B_ dual receptor antagonists as neuroprotective treatments for glaucomatous neuropathy.

**Electronic supplementary material:**

The online version of this article (doi:10.1186/s12868-017-0346-3) contains supplementary material, which is available to authorized users.

## Background

 Glaucoma is a common optic neuropathy characterized by dysfunction and degeneration of retinal ganglion cell (RGC) axons, optic nerve head cupping, and loss of RGCs, ultimately resulting in irreversible vision loss. Primary open angle glaucoma (POAG) is one of the most common types of glaucoma and is a slowly developing neurodegenerative disease. Progressing without pain or overt symptoms, POAG can cause significant neurodegeneration of RGCs and their axons before any visual deficits are discernible. More than 2 million Americans and approximately 60 million people worldwide are currently affected by glaucoma and based on statistical estimates that number is expected to reach 3 million patients in America and almost 80 million worldwide by the year 2020 [1]. Risk factors for developing glaucoma include age (over 60 years), myopia and race, with African-Americans being much more likely to develop POAG than Caucasians [2]. From the point of view of therapy, the most readily detectable and treatable risk factor for POAG is an elevation in intraocular pressure (IOP). Currently surgical and pharmacological treatments for POAG are primarily aimed at lowering IOP which have been shown to delay the progression of POAG. Since it is possible to develop glaucomatous neuropathy without an increase in IOP and given that in some patients POAG may still progress after treatment, it is important to identify other possible etiological contributors and treatment options for POAG.

One key contributor to the progression of glaucomatous optic neuropathy is the endothelin system of vasoactive peptides. Endothelins belong to a family of potent vasoactive peptides comprising of three isoforms: endothelin-1 (ET-1), endothelin-2 (ET-2), and endothelin-3 (ET-3) [3, 4]. The peptides act through two classes of G-protein coupled receptors: endothelin receptor A (ET_A_) and endothelin receptor B (ET_B_). Endothelins and their receptors have been shown to be present in multiple ocular tissues including the ciliary body, trabecular meshwork (TM) [5], retina [6, 7], and lamina cribrosa [8].

Previous reports have shown that ET-1 levels are elevated in human patients with primary open angle glaucoma [9–11], as well as in the congenital beagle model of glaucoma [12]. Endothelins have also been found to be elevated in the aqueous humor in the Morrison’s model of glaucoma [13] as well as in retinal microglia in the DBA/2J inherited model of glaucoma [14]. Although endothelin levels are shown to be elevated during glaucoma, direct evidence for the contribution of endothelin to glaucomatous neurodegeneration can be best assessed using animal models of glaucoma. Studies have shown that administration of ET-1 can produce degeneration of RGC axons in multiple animal models [15–19] and induce apoptosis of RGCs in rats [20].

A recent study in a mouse model showed activation of the endothelin system occurs early in glaucoma development before any observed morphological changes [14]. Administration of bosentan, a dual endothelin receptor antagonist (that blocks both ET_A_ and ET_B_ receptors) in the diet (100 mg/kg), was found to promote neuroprotection [14]. Previous studies from our laboratory demonstrated that following ocular hypertension, rats deficient in ET_B_ receptors showed a significant reduction in RGC loss compared to that seen in wild type rats [21]. However, the status of ET_A_ receptors under conditions of ocular hypertension and their precise role in neurodegeneration is not completely understood. The current study was aimed at understanding changes in ET_A_ receptor expression in the retinas of rats with elevated IOP.

## Methods

### Induction of ocular hypertension in adult Brown Norway rats

All animal procedures were carried out in accordance with the ARVO resolution for the Use of Animals in Ophthalmic and Vision Research. The protocol was approved by the Institutional Animal Care and Use Committee (IACUC) at the University of North Texas Health Science Center. For the current study, the Morrison’s model was used to induce ocular hypertension in adult male retired breeder Brown Norway rats as previously described [22]. Briefly, intraocular pressure was raised in one eye (by injecting approximately 50 µL of hypertonic saline through episcleral veins), while the other eye served as the contralateral control. Animals were sedated by intraperitoneal injection (100 µL/100 g body wt) of an anesthesia cocktail containing 55.6 mg/mL Ketamine (VEDCO), 5.6 mg/mL Xylazine (VEDCO), and 11.1 mg/mL Acepromazine (Lloyd Laboratories). After sedating the animals a small incision was made in the conjunctiva to expose the episcleral veins. A 1.8 M NaCl solution was then injected into an episcleral vein using a glass needle (TIP01TW1F, WPI) at a rate of 309 µL/min for 10 s. IOP was measured 2–3 times a week using a TonoLab tonometer (iCare, Finland). Six pressure readings were averaged for each IOP measurement and ten IOP measurements were obtained for each eye. IOP plots were generated from IOP values obtained from the surgically treated eye and contralateral control eye. The IOP exposure in each rat was computed by the integral product of the extent of IOP elevation and the number of days for which it maintained (expressed as mmHg-days). Typically, we get IOP exposures of approximately 61 to 90 mm Hg-days for 2 weeks of IOP elevation and 98 to 140 mm Hg-days for 4 weeks of IOP elevation.

### Immunohistochemistry

Animals were sacrificed by intraperitoneal pentobarbital injection (120 mg/kg body wt) and then eyes were carefully enucleated. A small incision (approximately 4–5 mm) was made using an ophthalmic micro surgical knife (MVR 20G, 160710, Cambrian-Medical) just posterior to the limbus and the eye was fixed in 4% PFA for 30 min. After fixation for 30 min the incision was continued around the eye until the entire anterior segment, including the lens, was completely removed. The posterior segment was fixed in 4% PFA for an additional 2.5 h. Eyes were then washed and placed in 70% ethanol until paraffin embedding. Sagittal retinal sections through the optic nerve (10 μm) were obtained using a microtome. Following deparaffinization, sections were blocked for 1 h in 5% normal donkey serum containing 5% BSA in PBS at room temperature. Sections were incubated in primary antibody and subsequently incubated with the appropriate secondary antibodies for 1 h each at room temperature. To detect any background staining, blank sections were prepared using the same protocol; however no primary antibody was added (Additional file 1: Figure S1, Additional file 2: Figure S2). Primary antibodies used were rabbit anti-ET_A_ (1:100, Sigma) and mouse anti-β-III-tubulin (1:500, Sigma). Secondary antibodies used were donkey anti-rabbit Alexa 647 (1:1000, Invitrogen) and donkey anti-mouse Alexa 488 (1:1000, Invitrogen). Sections were mounted and kept in Prolong^®^ Gold antifade reagent with DAPI (P36931, Invitrogen). Images were taken using a Zeiss LSM 510 META confocal microscope.

### Relative fluorescence intensity (RFI) measurements

#### Retina sections

An integrated projection of confocal z-stacks was created for every image and the fluorescence intensity was measured using ImageJ software. The freehand region of interest (ROI) tool was used to outline the regions of the retina to be analyzed. Bright field images were used for selecting ROIs. Once selected, ROIs were added to the ROI manager and overlaid onto the red channel (ET_A_ antibody) before measuring intensity. Values obtained for blank sections (no primary antibody) were averaged and served as background which was subtracted from the experimental values. After the background was subtracted, the measurements for each eye were averaged to obtain the mean fluorescence intensity. For each animal, the fluorescence intensity from contralateral (control) eyes were set to 1 to determine the relative change in ET_A_ receptor expression between IOP elevated and contralateral eyes.

#### Retinal ganglion cells

To measure expression of ET_A_ and ET_B_ receptors in primary RGCs confocal z-stack projections were generated for each image. The bright field images were used to visualize cells and the freehand ROI tool was used to trace somas which showed co-staining for β-III-tubulin (red fluorescence). Each trace was added to the ROI manager and overlaid onto the pink channel (ET_A_ and ET_B_ receptors) before measuring fluorescence intensity.

## 661W cell transfection and culture

For most cell culture experiments, transformed 661W photoreceptor cells were used. The 661W cells were grown in DMEM/low glucose media (Thermo Scientific) containing 10% fetal bovine serum (F2442, Sigma), 100 units/mL penicillin and 100 µg/mL streptomycin (SV30010, GE Healthcare). Stable clones overexpressing the ET_A_ receptor were generated by transfecting 661W cells with an ET_A_ receptor cDNA plasmid construct using Lipofectamine 2000 (11668-019, Invitrogen) and applying selection pressure with Geneticin (G418, 300 µg/mL) for 4 weeks. Briefly, cells were transfected with either pCMV6-Empty vector or pCMV6-ET_A_ expression vector (Origene, Rockville, MD) in DMEM serum-free media for 24 h and then placed in DMEM complete media for 24 h. Cells were dissociated by incubation in 0.0625% trypsin for 5 min at 37 °C and diluted to yield approximately 20 cells/mL and 100 µL of the cell suspension was added to multiple wells of a 96-well plate. Cells were then allowed to grow in DMEM complete media containing Geneticin (300 µg/mL) and were maintained for 4 weeks. Individual clones of 661W cells that displayed resistance to Geneticin were isolated, plated into separate wells of a 24-well plate and propagated further. Four stable clones were generated for both pCMV6-Empty and pCMV6-ET_A_ receptor expression vectors. ET_A_ receptor expression was confirmed by western blot and the clone yielding the highest level of ET_A_ receptor expression was used for further experiments.

### Live/dead assay

Stable clones of 661W cells expressing either empty vector or the ET_A_ receptor were counted using a hemocytometer and seeded in 24-well plates and grown to 50–60% confluence. Clones were treated with either DMEM serum-free media or DMEM serum-free media containing 100 nM ET-1 or ET-3 and allowed to incubate at 37 °C for 24 h. Following incubation, cells were washed twice using Dulbecco’s Phosphate Buffered Saline (DPBS) (#14278072, Gibco) and incubated with a Live/Dead assay kit (L3224, Life Technologies) for 30 min. Living cells were labeled green with Calcein AM and dead/dying cells were labeled red by Ethidium homodimer (EthD-1). Cell nuclei were labeled with a Hoechst stain to observe and obtain total cell numbers. Images were taken using a fluorescent microscope and cell counts were performed using ImageJ software (NIH, http://imagej.nih.gov/ij).

### MTT assay

MTT assays were performed using 661W cells stably overexpressing either the empty plasmid vector or ET_A_ cDNA encoding plasmid. A hemocytometer was used to count the cells and approximately 2000 cells were seeded in a black-wall clear-bottom 96 well plate. The cells were allowed to grow overnight at 37 °C. Before treatment, cells were washed once in DMEM serum-free media. Empty vector and ET_A_ vector expressing cells were then treated with 100 nM ET-1 or 100 nM ET-3 for 24 h. Following treatment, cells were washed once with DMEM serum-free media and then 20 μL of Promega CellTiter96 Aqueous One Solution was added to each well. The plate was incubated for 30 min and then the absorbance was measured at 490 nm for each well. Readings were taken using the Cytation5 (Bio-Tek). A standard curve was generated by measuring absorbance readings for wells seeded with 2000, 4000 and 8000 cells (Additional file 3: Figure S3). The standard curve was then used to extrapolate cell numbers from experimental absorbance readings.

### Fluorescence Assisted Cell Sorting (FACS) analysis

Stable clones overexpressing either the empty vector or the ET_A_ plasmid vector were used to assess differences in cell cycle by flow cytometry. Approximately 200,000 cells were seeded for each 100 mm dish for both Empty and ET_A_ expression vectors. Cells were allowed to reach 60% confluence and were treated with either 100 nM ET-1 or 100 nM ET-3 for 24 h in DMEM serum-free media. Untreated cells were also kept in DMEM serum-free media for 24 h. Following treatment, media was removed to collect any floating cells and spun down. Adherent cells were removed via trypsinization (0.0625%) for 5 min and spun down. The floating and adherent cell pellets were resuspended in 100 μL and 1 mL of PBS, respectively, and then combined. After adding the suspensions together 3.3 mL Ethanol was added to the cell suspension, mixed well, and stored overnight at −20 °C. The cells were spun down, resuspended in 500 μL PBS, and a cell count was performed. Approximately 500,000 cells were transferred to a flow cytometry tube (352058, BD Falcon) and the volume was made up to 500 μL with PBS. Cells were incubated with 2.5 μL of RNase A (20 mg/mL) for 30 min at 37 °C followed by 5 μL of propidium iodide (1 mg/mL) for 30 min at room temp. Cell cycle analysis was conducted using a Beckman Coulter Cytomics FC500 Flow Cytometry Analyzer.

### Western blot analysis

Stable clones overexpressing either the empty vector or the ET_A_ receptor plasmid vector were grown to confluence on 100 mm dishes. Cells were harvested using ice cold PBS and spun down at 3300 rpm for 5 min at 4 °C. Pellets were resuspended in an isotonic buffer (20 mM HEPES; 1 mM EDTA; 0.25 M sucrose; 0.5 mM PMSF, 1 mM DTT; 1× Halt protease inhibitor (78430, Thermo Scientific); 100 mM NaF; 1 mM Na_3_VO_4_. Cells were lysed by sonication and spun down at 12,000×*g* for 5 min at 4 °C. The supernatant was collected and spun down at 100,000×*g* for 45 min at 4 °C. The resulting pellet was then resuspended using an isotonic detergent buffer (20 mM HEPES; 1 mM EDTA; 0.25 M sucrose; 0.5 mM PMSF; 1 mM DTT; 1× Halt protease inhibitor; 0.1% Igepal CA 630; 0.1% Triton-X-100). Protein concentration was determined using spectrophotometry and 10–20 μg of protein was used for western blot experiments. Primary antibodies used to probe blots were rabbit anti-ET_A_ (1:1000; Sigma), rabbit anti-ET_B_ (1:10,000, Antibody Research Corporation), rabbit anti-Calnexin (1:1000, Cell Signaling) and mouse calnexin (1:1000, Cell Signaling). Secondary antibodies used were donkey anti-Rabbit HRP (1:10,000, GE Healthcare) and sheep anti-Mouse HRP (1:10,000, GE Healthcare). Blots were developed using SuperSignal™ West Dura extended duration substrate (34,075, Thermo Scientific).

### Adeno-associated virus production

Adeno-associated virus serotype 2 (AAV-2) encoding the ET_A_ receptor was generated in the lab by inserting ET_A_ cDNA (OriGene) into the AAV-2-IRES-hrGFP vector (Agilent Technologies, Santa Clara, CA). The restriction enzymes SalI-HF (New England Biolabs, Ipswich, MA) and XhoI (Promega, Madison, WI) were used to clone the ET_A_ cDNA fragment into the AAV-2-IRES-hrGFP vector. The resulting AAV-2-ET_A_ plasmid was sequenced (Lone Star Labs) to confirm the nucleotide sequence and ensure the cDNA was properly oriented. The AAV-2-IRES-hrGFP vector was used as control. The AAV-2-ET_A_ virus and AAV-2-GFP (control) viruses were then generated using AAV Helper-Free System according to the manufacturer’s protocol. Viral titer was determined using QuickTiter™ AAV Quantitation Kit (Cell Biolabs, Inc).

### Isolation and AAV-2 transduction of primary RGCs

Retinal ganglion cells were isolated and purified as previously described [23]. Briefly, RGCs were obtained from post-natal day 5 Sprague Dawley rat pups and purified by immunopanning. RGCs were positively selected for using the Thy1.1 antibody. Cells were seeded and grown in a 96-well plate (5000 cells/well) or 12-mm glass coverslips (30,000 cells/coverslip) and incubated in 10% CO_2_. RGCs were allowed to attach and produce neurites for 7 days prior to further experiments. The growth medium was changed every 3 days throughout the experiment.

#### Immunocytochemistry

Primary RGCs were seeded and grown on 12-mm glass coverslips. Seven days after seeding, AAV-2-GFP and AAV-2-ET_A_ was added to the cells and viral transduction was allowed to proceed for 11 days to permit robust expression of ET_A_ receptors. The growth medium was removed and cells were fixed using 4% PFA. After fixation, a permeablization buffer (0.1% sodium citrate, 0.1% Triton-X-100 in PBS) was added to each well for 5 min. Cells were incubated in blocking buffer (5% normal donkey serum, 5% bovine serum albumin in PBS) for 1 h at room temperature. Primary antibodies were diluted in antibody dilution buffer (1% BSA in PBS) and RGCs were incubated overnight at 4 °C. Primary antibodies used were rabbit anti-ET_A_ (1:100, Sigma), rabbit anti-ET_B_ (1:500 Antibody Research Company), mouse anti-β-III-tubulin (1:500, Sigma). Secondary antibodies were diluted in antibody dilution buffer and cells were incubated for 1 h at room temperature. Secondary antibodies used were donkey anti-rabbit Alexa 546 and donkey anti-mouse Alexa 647. Coverslips were mounted on slides using Prolong^®^ Gold antifade reagent with DAPI (P36931, Invitrogen). Images were taken with the Zeiss 510 Meta confocal microscope.

## Results

### Immunostaining for the ET_A_ receptor is increased in the retina of Brown Norway rats with elevated IOP

**Fig. 1:**
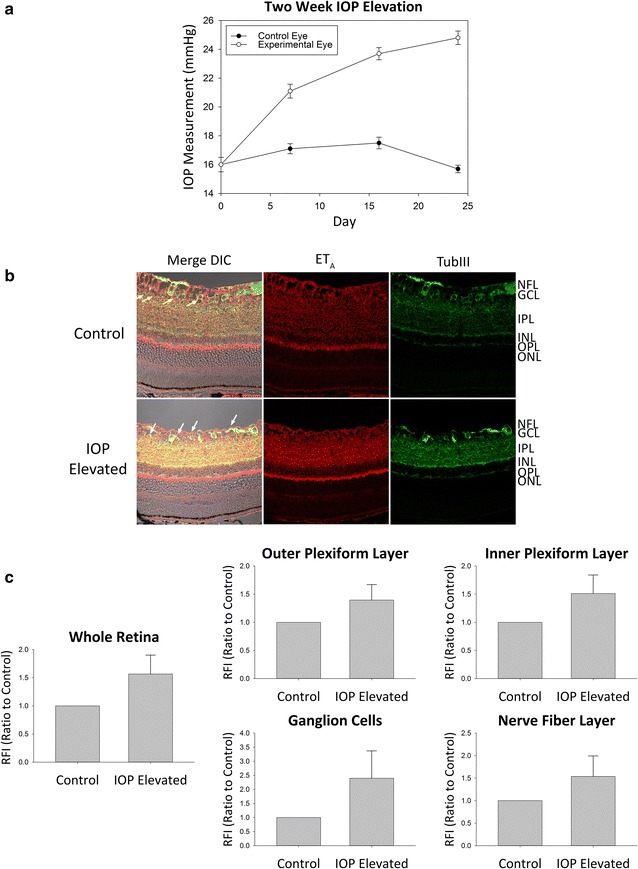
ET_A_ expression in retinas of adult Brown Norway rats following 2 week IOP elevation. **a** Representative graph of IOP measurements for IOP elevated (*white circles*) and contralateral control (*black circles*) eyes in adult male Brown Norway rats. **b** Representative images. Immunostaining of retina sections probed for ET_A_ receptors (*red fluorescence*) and β-III-tubulin (*green fluorescence*) following 2 weeks of IOP elevation. *Arrows* point to RGCs. **c** Relative fluorescent intensity for the retina, OPL, IPL, GCs and NFL. *Bars* represent mean ± SEM (n = 7 animals/group). *ONL* outer nuclear layer, *OPL* outer plexiform layer, *INL* inner nuclear layer, *IPL* inner plexiform layer, *GCL* ganglion cell layer, *NFL* nerve fiber layer, *TubIII* β-III-tubulin

Our laboratory has previously demonstrated that the endothelin receptor B (ET_B_) is upregulated following an increase in IOP and contributes to the degeneration of RGCs [21]. The focus of the present study was to determine if similar changes in the ET_A_ receptor expression was observed after IOP elevation. Following 2 weeks of IOP elevation in Brown Norway rats, retinas obtained from both IOP elevated and contralateral control eyes showed immunoreactivity for ET_A_ receptors in multiple retinal layers including the nerve fiber layer (NFL), ganglion cell layer (GCL), inner plexiform layer (IPL), and outer plexiform layer (OPL) (Fig. 1b). Compared to the contralateral controls, a qualitative analysis of retinas obtained from IOP elevated eyes showed an increasing trend in ET_A_ receptor expression within the OPL, IPL and GCL. Expression of ET_A_ receptors was colocalized with RGCs using β-III-tubulin which is a selective neuronal marker (Fig. 1b). Additionally, a modest increase in immunostaining for ET_A_ receptors was also observed in the NFL compared to the contralateral controls. A semi-quantitative analysis measuring the change in fluorescence intensity was also performed using ImageJ software. Projections from confocal image z-stacks were analyzed for relative fluorescence intensity of ET_A_ receptor expression in IOP elevated eyes and corresponding contralateral control eyes to determine fold change. While five out seven rats showed an increase in immunostaining for the ET_A_ receptors, two of the seven rats showed a slight decrease, thereby did not attain statistical significance at the 2 week time point of IOP elevation (retina, RFI = 1.57 ± 0.34; OPL, RFI = 1.40 ± 0.28; IPL, RFI = 1.51 ± 0.33; GCs, RFI = 2.40 ± 0.98; NFL, RFI = 1.54 ± 0.46), however there was a clear trend towards an increase in immunostaining for ET_A_ receptors (Fig. 1c).

**Fig. 2:**
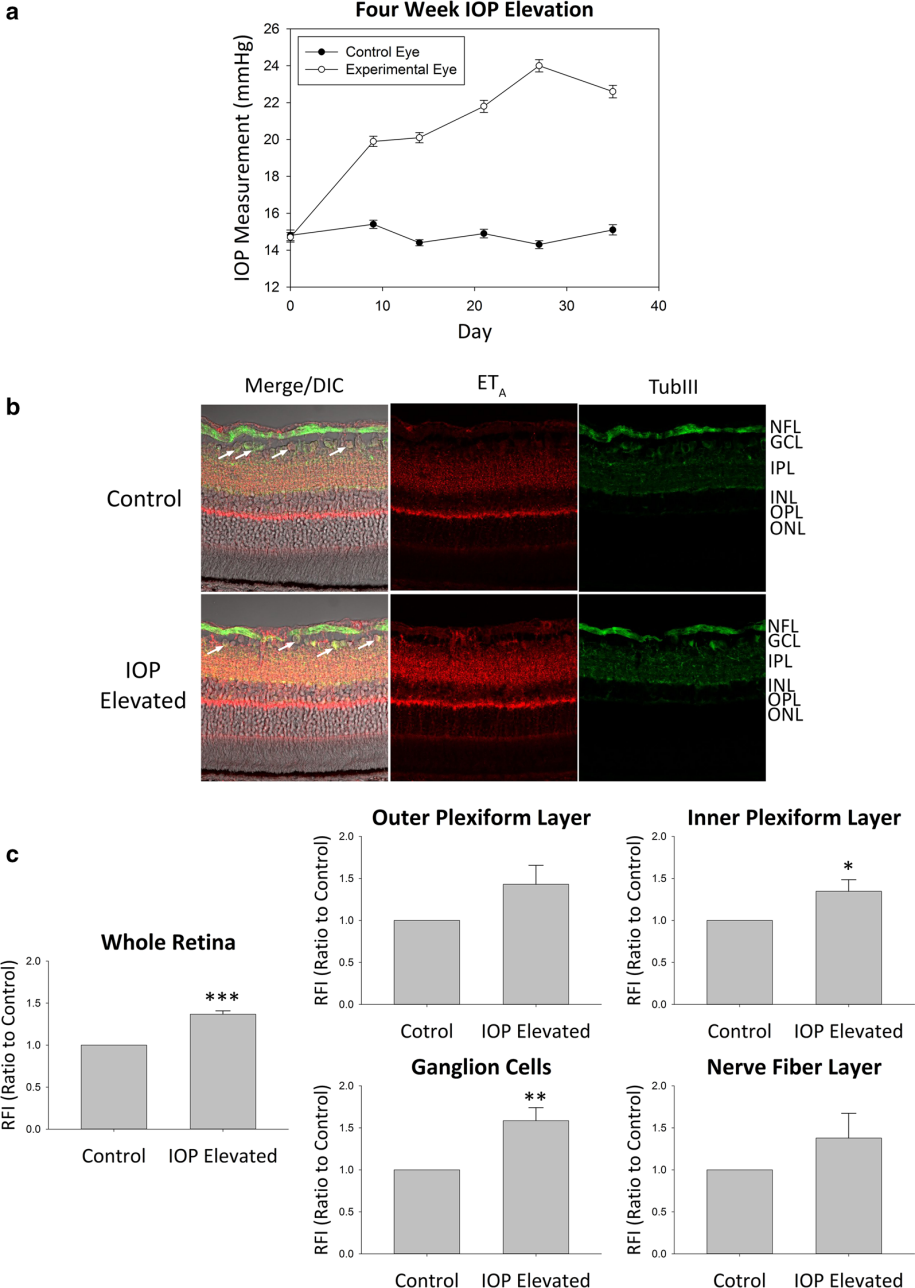
ET_A_ expression in retinas of adult Brown Norway rats following 4 week IOP elevation. **a** Representative graph of IOP measurements for IOP elevated (*white circles*) and contralateral control (*black circles*) eyes in adult male Brown Norway rats. **b** Representative images. Immunostaining of retina sections probed for ET_A_ receptors (*red fluorescence*) and β-III-tubulin (*green fluorescence*) following 4 weeks of IOP elevation. *Arrows* point to RGCs. **c** Relative fluorescent intensity for the retina, OPL, IPL, GCs and NFL. *Bars* represent mean ± SEM (n = 4 animals/group). *Asterisks* indicate statistical significance *p < 0.05; **p < 0.01; ***p < 0.001 by student’s t-test. *ONL* outer nuclear layer, *OPL* outer plexiform layer, *INL* inner nuclear layer, *IPL* inner plexiform layer, *GCL* ganglion cell layer, *NFL* nerve fiber layer, *TubIII* β-III-tubulin

Similar to the pattern of expression observed at 2 weeks of IOP elevation, increased immunoreactivity for ET_A_ receptor in the retina was also observed in the NFL, GCL, IPL, and OPL (Fig. 2b). Retinas obtained from Brown Norway rats following 4 weeks of IOP elevation qualitatively showed the greatest increase in immunoreactivity for ET_A_ receptors in the IPL and GCL. A faint increase in ET_A_ receptor expression was also observed in the NFL and OPL. A semi-quantitative analysis was performed which indicated a statistically significant increase in ET_A_ receptor expression overall in the retina (RFI = 1.37 ± 0.04, n = 4, p < 0.001) as well as in the IPL (RFI = 1.35 ± 0.14, n = 4, p < 0.05) and GCs (RFI = 1.59 ± 0.16, n = 4, p < 0.01) following 4 weeks of IOP elevation (Fig. 2c). Statistical significance was not reached in either the OPL (RFI = 1.43 ± 0.23) or the NFL (RFI = 1.38 ± 0.30) however the same trend of increased ET_A_ receptor expression was still present. Taken together, the data suggest that ET_A_ receptor expression was upregulated due to ocular hypertension in rats and that the increase in ET_A_ receptor expression was sustained during longer durations of IOP elevation.

### Stable overexpression of the ET_A_ receptor produces an increase cell death in 661W cells

**Fig. 3:**
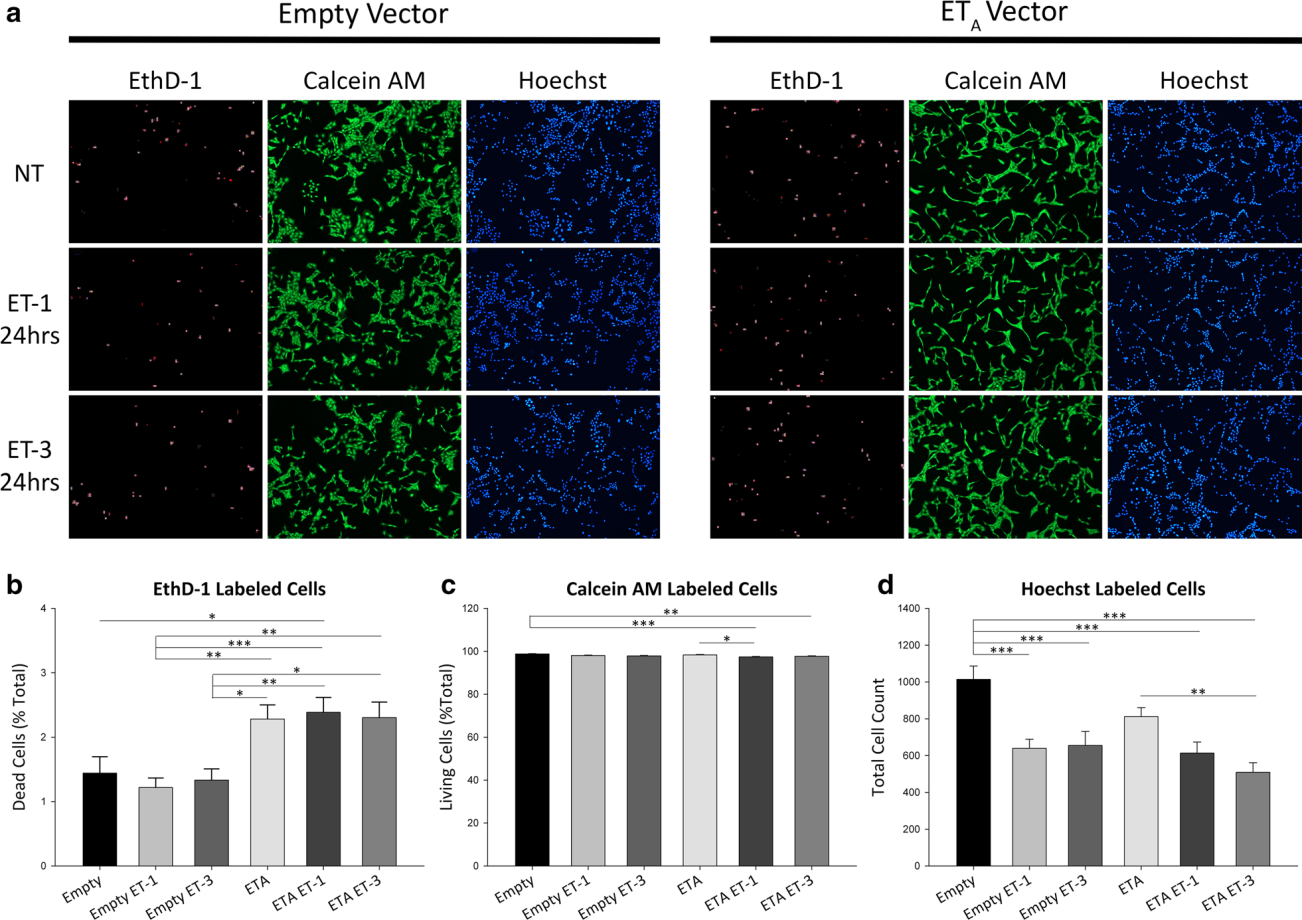
Live-Dead assay of 661W cells stably overexpressing the ET_A_ receptor. **a** Representative images of EthD-1 (*red fluorescence*), calcein AM (*green fluorescence*) and Hoechst (*blue fluorescence*) labeled 661W stable clones indicating dead, living and total cells respectively. Images were taken following 24 h treatment with either 100 nM ET-1 or ET-3. **b**–**d** Quantitative analysis of **b** EthD-1+, **c** calcein AM+ and **d** Hoechst labeled cells. *Bars* represent mean ± SEM (n = 3). *Asterisks* indicated statistical significance *p < 0.05; **p < 0.01; ***p<0.001 by one-way ANOVA and Tukey’s post hoc test

In order to elucidate the role of ET_A_ receptor in the retina and its possible contribution to neurodegeneration, stable clones were generated by transfecting 661W photoreceptor cells with either an ET_A_ receptor cDNA vector or the corresponding empty plasmid vector. A live/dead assay using Calcein AM and Ethidium homodimer-1 (EthD-1) was performed with the stable ET_A_ receptor clone and the empty vector clone following a 24 h treatment with either 100 nM ET-1 or ET-3. Cells were also labeled using Hoechst nuclear stain for visualization and quantitation of total cell numbers (Fig. 3a). Analysis of EthD-1+ cells (dead/dying cells, red fluorescence) in empty vector transfected group revealed no change in the percentage of dead/dying cells following treatment with either 100 nM ET-1 (1.22 ± 0.15%) or ET-3 (1.33 ± 0.17%) compared to untreated cells (1.44 ± 0.25%) (Fig. 3b). Stable clones overexpressing the ET_A_ receptor, regardless of treatment group, showed an increase in the percentage of dead/dying cells when compared to empty vector clones. There was no further exacerbation of cell death in ET_A_ vector expressing cells (2.28 ± 0.22%) following treatment of either 100 nM ET-1 (2.39 ± 0.23%) or ET-3 (2.30 ± 0.24%) (Fig. 3b).

In addition to quantifying the percentage of dead/dying cells, Calcein AM+ cells (living, green fluorescence) were also analyzed. No decrease in the percentage of living cells was observed in empty vector cells (98.77 ± 0.18%) following 24 h treatment with either 100 nM ET-1 (98.03 ± 0.21%) or 100 nM ET-3 (97.85 ± 0.21%) (Fig. 3c). The percentage of living ET_A_ receptor overexpressing cells (98.33 ± 0.22%) was reduced after treatment with ET-1 (97.37 ± 0.21%, n = 3, p < 0.05) but not with ET-3 (97.69 ± 0.24%) (Fig. 3c). Using Hoechst staining to quantify total cells in each treatment group, we consistently found a lesser number of total cells in wells containing ET_A_ overexpressing cells (811.89 ± 48.56) compared to empty vector cells (1013.94 ± 72.91) (Fig. 3d). Total cell number was further reduced in empty vector cells after treatment with either ET-1 (639.83 ± 48.75, n = 3, p < 0.001) or ET-3 (655.12 ± 76.45, n = 3, p < 0.001). Cell numbers for the ET_A_ vector transfected group were further decreased following ET-1 treatment (613.42 ± 60.37) and reached statistical significance in the ET-3 treatment group (508.94 ± 52.50, n = 3, p < 0.01) (Fig. 3d).

### ET_A_ receptor overexpression in 661W cells results in decreased cell viability

**Fig. 4:**
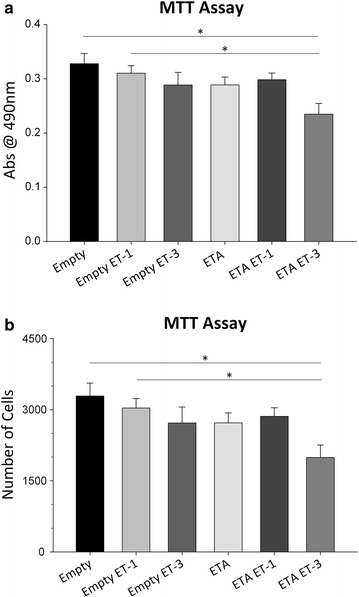
ET_A_ receptor overexpression reduces overall cell viability in 661W cells. **a** Quantitative analysis of MTT assay absorbance readings at 490 nm for empty vector and ET_A_ receptor vector stable clones following 24 h treatment with either 100 nM ET-1 or ET-3. **b** Graph of interpolated cell counts generated from a standard curve and experimental absorbance readings. *Bars* represent mean ± SEM (n = 20). *Asterisks* indicated statistical significance *p < 0.05 by one-way ANOVA and Tukey’s post hoc test

To clarify the above findings, an additional cell viability/proliferation assay was performed. To confirm that ET_A_ overexpression compromises cell viability, an MTT (3-(4,5-dimethylthiazol-2-yl)-2,5-diphenyltetrazolium bromide) cell proliferation assay was performed on empty vector and ET_A_ cDNA overexpressing 661W cells. Absorbance readings at 490 nm were taken for each well of a 96-well plate. Empty vector transfected cells treated with 100 nM ET-1 (Abs = 0.310 ± 0.014) or 100 nM ET-3 (Abs = 0.289 ± 0.023) for 24 h showed a decreasing trend in absorbance readings compared to untreated empty vector cells (Abs = 0.328 ± 0.019) though not statistically significant (Fig. 4a). Absorbance readings for cells overexpressing the ET_A_ receptor (Abs = 0.289 ± 0.015) did not show a significant decrease compared to empty vector cells. The greatest reduction in absorbance was observed in ET_A_ vector cells treated with 100 nM ET-3 (Abs = 0.235 ± 0.020) for 24 h, however, no change was detected after treatment with ET-1 (Abs = 0.298 ± 0.013), compared to the untreated ET_A_ receptor transfected cells (Fig. 4a). A standard curve generated from absorbance readings from known cell numbers was used to extrapolate the number of cells in the experimental groups (Fig. 4b).

These results are in line with the live/dead assay and which shows a decrease in cell viability for cells overexpressing the ET_A_ receptor. Interestingly in both assays, the increase in cell death was most prominent following treatment with ET-3, an ET_B_ receptor agonist.

### Cell cycle kinetics is altered by stable overexpression of the ET_A_ receptor in 661W cells

After demonstrating that ET_A_ receptor overexpression affected cell viability in the 661W stable clone, we wanted to further explore the effect of ET_A_ receptor overexpression on the cell cycle. Fluorescence-Assisted Cell Sorting (FACS) analysis using propidium iodide (PI) was utilized to observe if there were alterations in certain phases of the cell cycle between empty vector and ET_A_ receptor overexpressing cells.

**Fig. 5:**
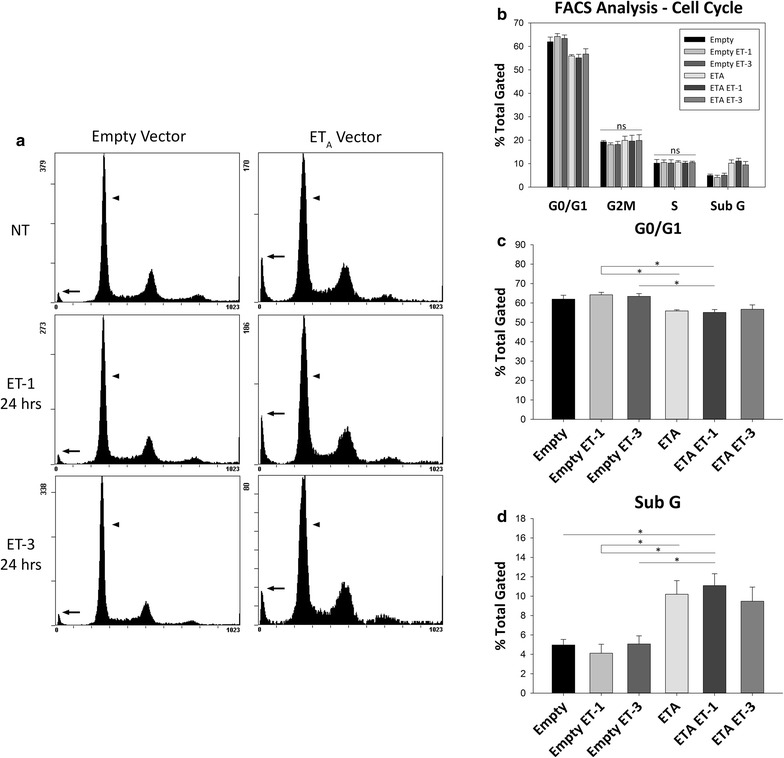
ET_A_ receptor overexpression alters cell cycle in 661W cells. **a** Representative images. FACS analysis of cell cycle in empty and ET_A_ receptor vector 661W stable clones following 24 h treatment with either 100 nM ET-1 or ET-3. *Arrows* show Sub G peak; *arrowheads* show G0/G1 peak. **b**–**d** Quantitation of **b** cell cycle phases, **c** G0/G1 phase and **d** Sub G phase. *Bars* represent mean ± SEM (n = 3). *Asterisks* indicated statistical significance *p < 0.05, by one-way ANOVA and Tukey’s post hoc test. *ns* no statistical significance

Analysis of the histogram plots revealed differences in the G0/G1 phase peak (arrowheads) and the Sub G phase peak (arrows) between empty vector and ET_A_ receptor vector cells (Fig. 5a). No difference was found in the G2M and S phases for empty or ET_A_ receptor vector cells (Fig. 5b). There was no change in the G0/G1 peak for empty vector cells after treatment with either 100 nM ET-1 or ET-3 (empty: 61.96 ± 2.04%; empty ET-1: 64.23 ± 1.27%; empty ET-3: 63.41 ± 1.46%) or in ET_A_ vector cells following treatment (ET_A_: 55.90 ± 0.62%; ET_A_ ET-1: 55.12 ± 1.52%; ET_A_ ET-3: 56.75 ± 2.27%) (Fig. 5c). Statistical significance was reached in G0/G1 phase between empty vector ET-1 treated cells versus ET_A_ vector ET-1 treated cells (64.23 ± 1.27 vs. 55.12 ± 1.52%, n = 3, p < 0.05) and empty vector ET-1 treated cells versus ET_A_ vector cells (64.23 ± 1.27 vs. 56.75 ± 2.27%, n = 3, p < 0.05). A significant difference was also seen in empty vector ET-3 treated cells versus ET_A_ vector ET-1 treated cells (63.41 ± 1.46 vs. 55.12 ± 1.52%, n = 3, p < 0.05) (Fig. 5c).

Assessment of the Sub G peak also showed no difference between empty vector cells following ET-1 or ET-3 treatment (empty: 4.96 ± 0.57%; empty ET-1: 4.11 ± 0.93%; empty ET-3: 5.07 ± 0.84%) or between ET_A_ vector cells following treatment (ET_A_: 10.20 ± 1.42%; ET_A_ ET-1: 11.09 ± 1.23%; ET_A_ ET-3: 9.47 ± 1.46%) (Fig. 5d). There was a trend towards an increase of the SubG peak in ET_A_ vector cells compared to empty vector cells although it did not reach statistical significance (10.20 ± 1.42 vs. 4.96 ± 0.57%, n = 3). Significance was reached however between ET_A_ vector ET-1 treated cells and empty vector cells (11.09 ± 1.23 vs. 4.96 ± 0.57%, n = 3, p < 0.05). A difference was also observed between empty vector ET-1 treated cells and ET_A_ vector cells (4.11 ± 0.93 vs. 10.20 ± 1.42%, n = 3, p < 0.05) as well as ET_A_ vector ET-1 treated cells (4.11 ± 0.93 vs. 11.09 ± 1.23%, n = 3, p < 0.05). Empty vector cells treated with ET-3 also showed a significant difference from ET_A_ vector ET-1 treated cells (5.07 ± 0.84 vs. 11.09 ± 1.23%, n = 3, p < 0.05) (Fig. 5d).

### Overexpression of ET_A_ receptors upregulate ET_B_ receptor expression in 661W cells

**Fig. 6:**
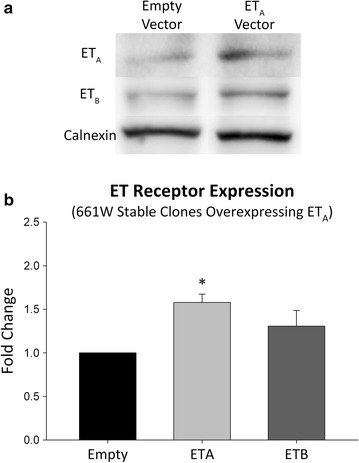
Overexpression of the ET_A_ receptor can increase in ET_B_ receptor expression. **a** Representative images. Immunoblot showing the expression of ET_A_ and ET_B_ receptors in the plasma membrane fraction of 661W stable clones overexpressing the ET_A_ receptor. **b** Immunoblot densitometry showing fold change in protein expression of both ET_A_ and ET_B_ receptors in 661W cells overexpressing the ET_A_ receptor compared to empty vector cells. Protein normalized to calnexin expression. *Bars* represent mean ± SEM (n = 5). *Asterisks* indicated statistical significance *p < 0.05 by student’s t-test

Considering that the ET-3 peptide is selective for the ET_B_ receptor and has low affinity for the ET_A_ receptor it was interesting to see decreased cell viability in ET_A_ receptor overexpressing cells treated with ET-3 (Figs. 3, 4). To explain how ET_A_ receptor overexpressing cells treated with the ET-3 peptide showed a reduction in total cell numbers in both the live/dead assay and the MTT assay, we wanted to know if there were changes in the expression of ET_B_ receptors. To determine this, we performed an immunoblot analysis to determine changes in ET_B_ receptor expression in cells overexpressing ET_A_ receptors. ET_B_ receptor expression was found to be variable in cells overexpressing the ET_A_ receptor. In some samples ET_B_ expression was relatively unchanged while in other samples there was an almost twofold increase in expression. Overall, there was an increasing trend in ET_B_ receptor levels in 661W cells stably overexpressing the ET_A_ receptor (Fig. 6).

### ET_A_ receptor overexpression increases cell death in primary RGCs

**Fig. 7:**
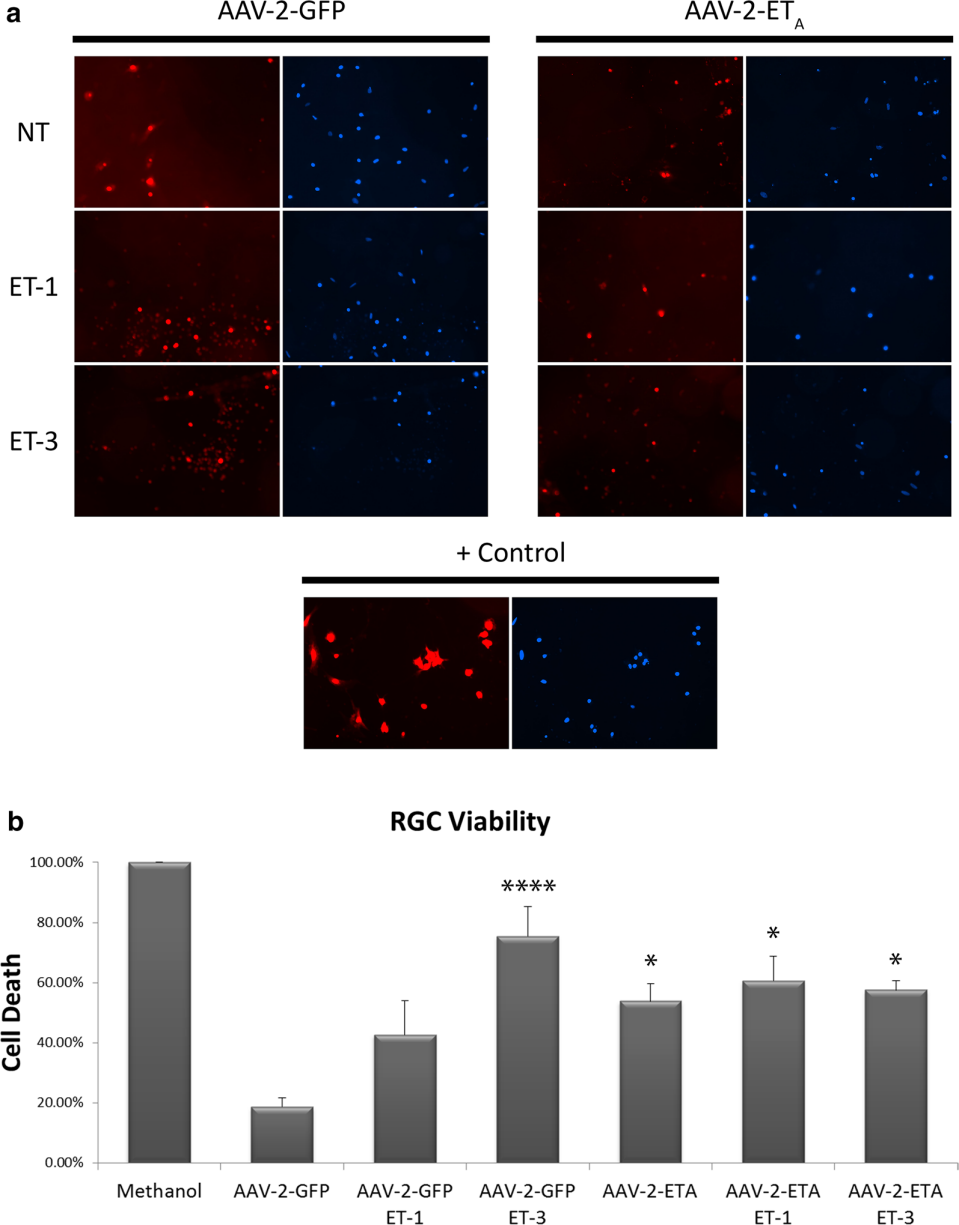
ET_A_ receptor overexpression increases cell death in primary RGCs. **a** Representative images of dead (*red*) and total (*blue*) RGCs following transduction with either AAV-2-GFP or AAV-2-ET_A_ and 24 h treatment with 100 nM ET-1 or ET-3. Positive control shows RGCs treated with methanol. **b** Quantitative analysis of percent cell death of RGCs. *Bars* represent mean ± SEM (n = 3). *Asterisks* indicated statistical significance *p < 0.05, ****p < 0.0001 by one-way ANOVA and Tukey’s post hoc test

Since the above data was collected from the 661W cell line, we wanted to determine if ET_A_ receptor overexpression showed the same increase in cell death of primary RGCs. To determine this, primary RGCs were transduced with either the AAV-2-GFP or AAV-2-ET_A_ virus and subsequently treated with ET-1 (100 nM) or ET-3 (100 nM). Cell viability was determined by treating for 30 min with the NUCLEAR-ID^®^ Blue/Red cell viability reagent (Enzo Life Sciences). Cell death of RGCs transduced with AAV-2-GFP vector without endothelin treatment was 18.66 ± 0.03% (Fig. 7). Following 24 h treatment with 100 nM ET-1 or 100 nM ET-3, cell death of AAV-2-GFP transduced RGCs was increased to 42.69 ± 0.11 and 75.45 ± 0.10% (p < 0.0001), respectively. RGCs transduced with the AAV-2-ET_A_ vector showed significantly greater cell death, 53.86 ± 0.06% (p < 0.05), compared to AAV-2-GFP transduced RGCs. Unlike AAV-2-GFP transduced RGCs, no exacerbation of cell death was observed in AAV-2-ET_A_ transduced RGCs after 24 h treatment with either 100 nM ET-1 (60.57 ± 0.08%) or 100 nM ET-3 (57.53 ± 0.03%). Similar to that observed in 661W cells, total RGC numbers were decreased following treatment with ET-1 or ET-3 and in RGCs transduced with AAV2-ET_A_.

### ET_A_ receptor overexpression increases ET_B_ receptor expression in primary RGCs

**Fig. 8:**
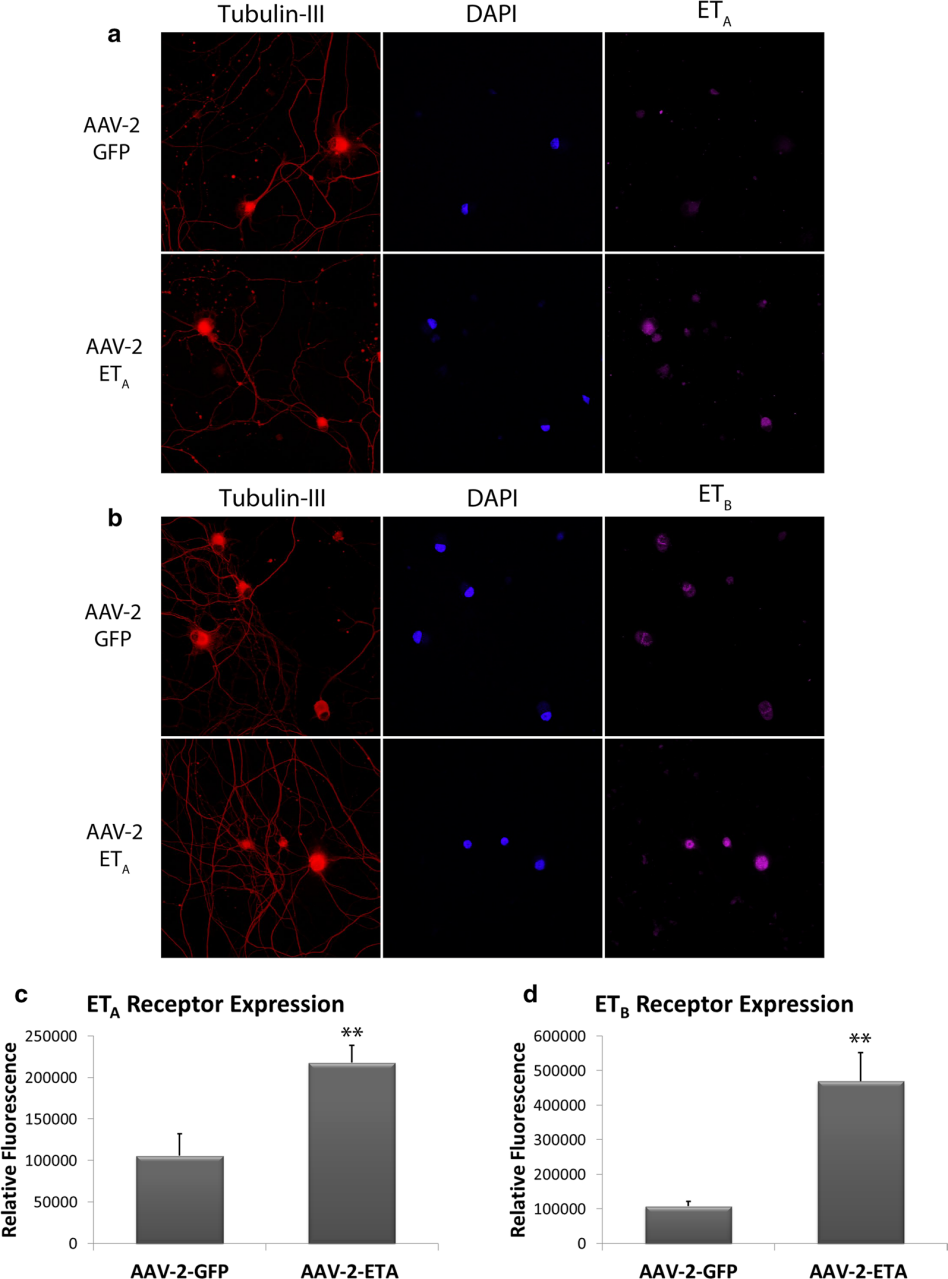
Overexpression of the ET_A_ receptor increases ET_B_ receptor expression in primary RGCs. **a**, **b** Representative images. Immunostaining showing the expression of ET_A_ and ET_B_ receptors in purified RGCs following transduction with either AAV-2-GFP or AAV-2-ET_A_. β-III-tubulin was used as a selective marker of RGCs. DAPI staining was used to visualize nuclei of RGCs. **c**, **d** Quantitative analysis of ET_A_ receptor (**c**) and ET_B_ receptor (**d**) expression in purified RGCs. *Bars* represent mean ± SEM (n = 3). *Asterisks* indicate statistical significance **p < 0.01 by student’s t-test

Primary RGCs transduced with the AAV-2-ET_A_ virus showed a 2.1 fold increase in ET_A_ receptor expression (p < 0.001) compared to cell transduced with the AAV-2-GFP virus (Fig. 8c). Immunostaining for the ET_B_ receptor revealed a 4.4 fold increase in ET_B_ receptor expression (p < 0.001) after transduction with AAV-2-ET_A_ compared to AAV-2-GFP (Fig. 8d) .

## Conclusions

The endothelin system has been established to have a neurodegenerative role in animal models of primary open angle glaucoma. Most of the focus in the literature has been on the ET_B_ receptor, with previous studies from our lab demonstrating that the ET_B_ receptor is a major contributor to glaucomatous neurodegeneration [21]. Although there has been very few studies focusing on the ET_A_ receptor in glaucoma, some reports have alluded to a possible role for the ET_A_ receptor in neurodegeneration.

Many reports indicate that one of the earliest site of damage during glaucoma is in the axons of RGCs at the optic nerve head suggestive of an axonopathy. Studies assessing axonal transport in the optic nerve following ET-1 administration found a decrease in the transport of mitochondrial subcomponents [24] and a disruption of the transport and delivery of fast axonal transport cargos [25]. At the optic nerve head ET-1 induces an increase in ET_B_ receptors in optic nerve head astrocytes [26] and induces proliferation of astrocytes; however the proliferative antagonist was blocked using either an ET_A_ or ET_B_ antagonist [27] as well as the ET_A_/ET_B_ dual antagonist. These studies suggest that the endothelin peptides have the ability to induce neurodegenerative effects by numerous cellular mechanisms involving both ET_A_ and ET_B_ receptors. Howell et al. [28] found an increase in mRNA expression of both ET_A_ and ET_B_ receptors at an early stage of disease progression in the DBA/2J mouse model of glaucoma. In addition, our lab has shown that ET_A_ mRNA levels are also increased in the retina following IOP elevation in Brown Norway rats [29]. Furthermore, recent studies have shown significant neuroprotection using a dual endothelin antagonist [14, 28]. These findings suggest that in addition to the ET_B_ receptor, the ET_A_ receptor could also play a role in glaucomatous neurodegeneration; however the precise role of the ET_A_ receptor is largely unknown.

In this study we have demonstrated for the first time, by immunohistochemistry. that there is an increase in ET_A_ receptor expression in multiple layers of the retina following 2- and 4 weeks of IOP elevation, compared to the corresponding contralateral control eyes in Brown Norway rats. We further demonstrated by multiple in vitro techniques that ET_A_ receptor overexpression leads to an overall decrease in cell viability of both 661W cells and primary RGCs. The cell death observed in 661W cells was minimal, possibly due to confounding effects of the cell line which is inherently proliferative, thereby attenuating the cell death inducing effect of endothelins. The ability of endothelins to promote cell death was clearly evident in the primary RGCs which do not proliferate since they are terminally differentiated. While ET_A_ overexpression in primary RGCs produced increased cell death, there was no further exacerbation of cell death after treatment of these cells with ET-1 and ET-3. This suggests that there was a threshold of endothelin receptor activation following ET_A_ receptor overexpression, possibly due to autocrine effects mediated by endothelin release from RGCs. In 661W cells stably overexpressing the ET_A_ receptor, we observed a greater reduction in cell viability when treated with ET-3 than when treated with ET-1 Considering that ET-3 is an ET_B_ agonist which has low affinity for the ET_A_ receptor, it was more likely that cell death was occurring due to ET_A_ receptor mediated upregulation of the ET_B_ receptor. To support this conclusion we performed an immunoblot analysis of 661W cells stably overexpressing ET_A_ receptors and found an increasing trend in ET_B_ receptors expression (not significant), compared to empty vector cells. In primary RGCs, immunostaining revealed AAV-2 mediated overexpression of the ET_A_ receptor produced a greater than fourfold increase in ET_B_ receptor expression. In support of these findings, He et al. [30] showed that treatment of RGCs with either ET-1 or ET-3 produced an increase in both ET_A_ and ET_B_ receptor proteins detected by immunocytochemistry. Our findings, in this study and in conjunction with previous studies, have provided evidence that both ET_A_ and ET_B_ receptors are upregulated due to an elevation in IOP in Brown Norway rats. It is generally accepted that the ET_B_ receptor is the major contributor to cell death of RGCs [21], however based upon our current findings, the involvement of the ET_A_ receptor in the upregulation of ET_B_ receptors, thereby contributing to cell death, is a plausible scenario (Figs. 6, 8).

In animal models of traumatic brain injury an ET_A_ receptor antagonist showed the ability to limit neuronal damage [31]. Pretreatment with BQ-123, an ET_A_ receptor antagonist significantly reduced axonal injury and improved retention of cognitive scores [32]. While the cellular and molecular mechanisms involved in neuroprotection in the brain and the retina might not be exactly the same, the greater neuroprotective effects of dual endothelin receptor antagonists, compared to ET_B_ receptor selective inhibitors alone, suggests the involvement of both receptors in neurodegeneration.

While it is evident that the endothelin receptors are upregulated during IOP elevation, some questions still need to be addressed in the future. One such question is how does an increase in IOP lead to elevated endothelin levels and increased endothelin receptor expression? The second question that still remains to be answered is what pathways are involved during endothelin receptor mediated neurodegeneration? One study showed that when shear stress is applied to endothelial cells there is an increase in ET_B_ receptors, c-jun, and AP-1 [33] and a study using a rodent model of glaucoma also showed involvement of AP-1 and C/EBPβ in the upregulation of ET_B_ receptors [29], although the mechanotransduction pathways have not been fully elucidated. Interestingly, following treatment of RGCs with either ET-1 or ET-3 we found an increase in both c-Jun and phospho-c-Jun suggesting that the JNK/c-Jun pathway may be a contributor to endothelin receptor upregulation and endothelin-mediated cell death of RGCs. Assessment of the contribution of both endothelin receptors to glaucomatous neurodegeneration provides a good rationale for developing endothelin antagonists as neuroprotective agents for the treatment of glaucoma.

## Additional files

**Additional file 1: Figure S1.** Immunostaining negative control for 2 week IOP elevated retina sections. No primary antibody for endothelin A (ET_A_) receptor or β-III-tubulin (TubIII) was added. DIC image show retinal layers.

**Additional file 2: Figure S2.** Immunostaining negative control for 4 week IOP elevated retina sections. No primary antibody for endothelin A (ET_A_) receptor or β-III-tubulin (TubIII) was added. DIC image show retinal layers.

**Additional file 3: Figure S3.** Standard curve generated from the averaged absorbance readings at 490 nm of two-thousand, four thousand and eight thousand 661W cells. Points represent average absorbance ± SEM at each cell density (n = 20).

## Abbreviations

POAG: primary open angle glaucoma
IOP: intraocular pressure
ET_A_: endothelin A receptor
ET_B_: endothelin B receptor
ET-1: endothelin-1
ET-3: endothelin-3
NFL: nerve fiber layer
GCL: ganglion cell layer
IPL: inner plexiform layer
OPL: outer plexiform layer
