# Supplementary figures and images for: Upregulation of the endothelin A (ETA) receptor and its association with neurodegeneration in a rodent model of glaucoma
Source: BMC Neurosci. 2017 Mar 1;18:27. doi: 10.1186/s12868-017-0346-3 (PMC5333388; doi:10.1186/s12868-017-0346-3)

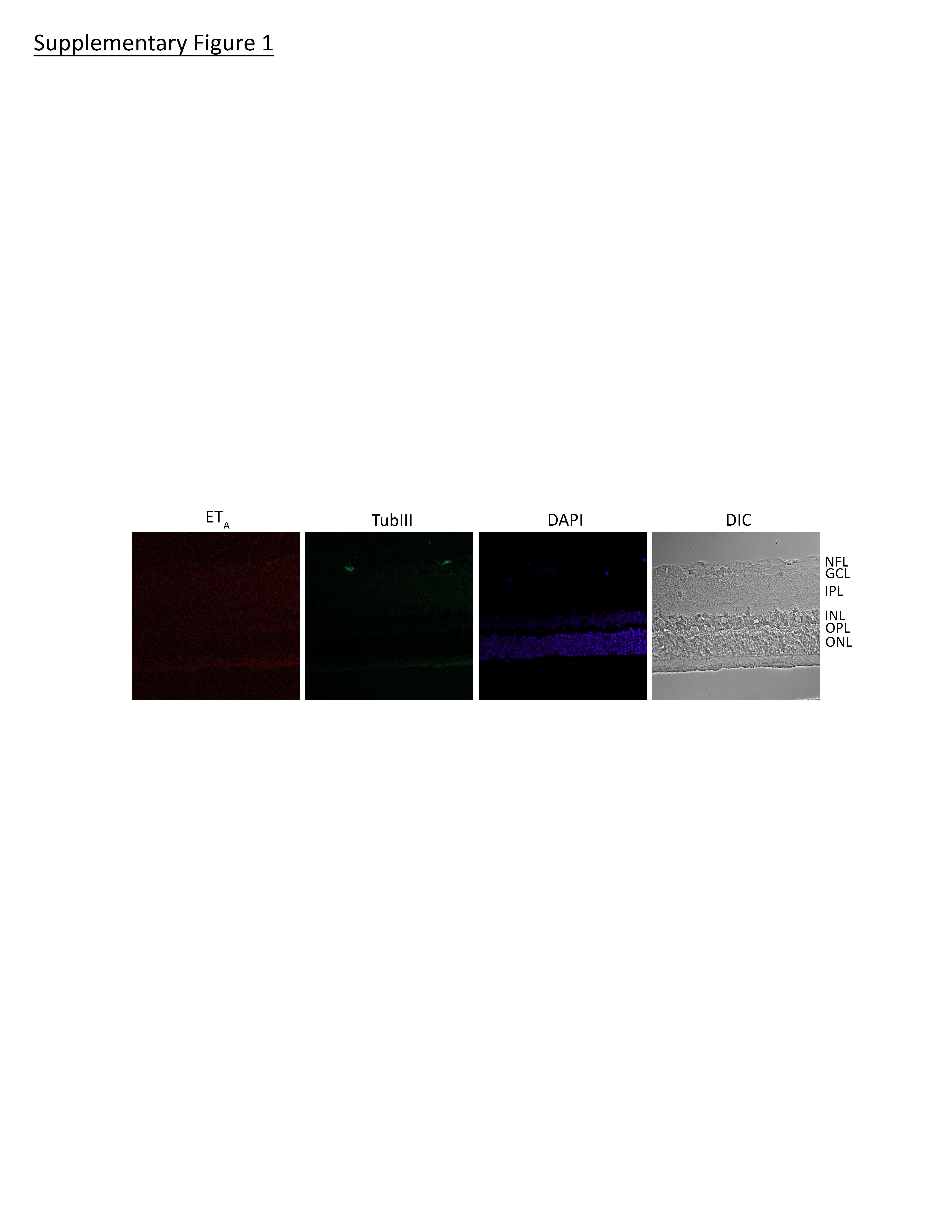

Supplement: Supplementary file 1 — Additional file 1: Figure S1. Immunostaining negative control for 2 week IOP elevated retina sections. No primary antibody for endothelin A (ETA) receptor or β-III-tubulin (TubIII) was added. DIC image show retinal layers. [file 12868_2017_346_MOESM1_ESM.png]

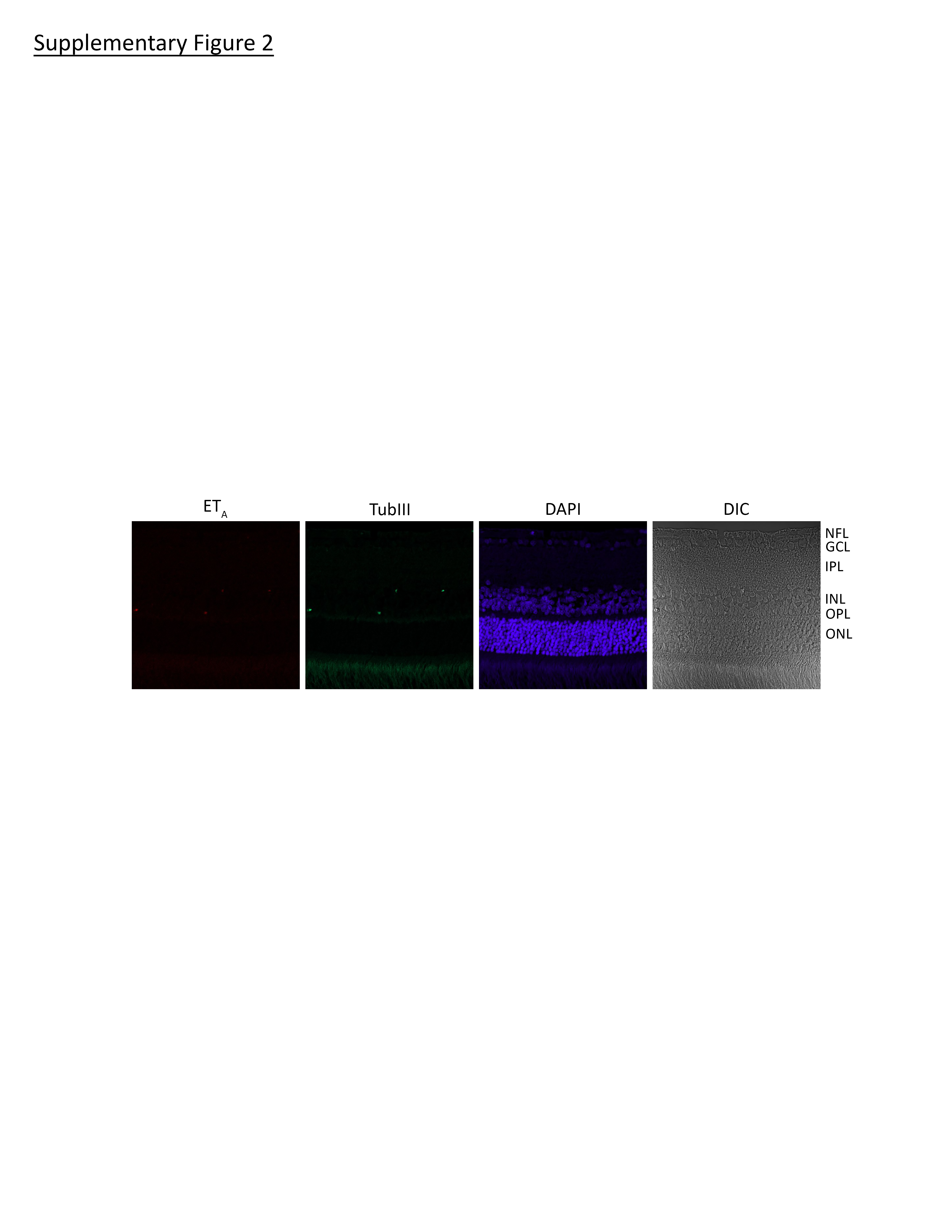

Supplement: Supplementary file 2 — Additional file 2: Figure S2. Immunostaining negative control for 4 week IOP elevated retina sections. No primary antibody for endothelin A (ETA) receptor or β-III-tubulin (TubIII) was added. DIC image show retinal layers. [file 12868_2017_346_MOESM2_ESM.png]

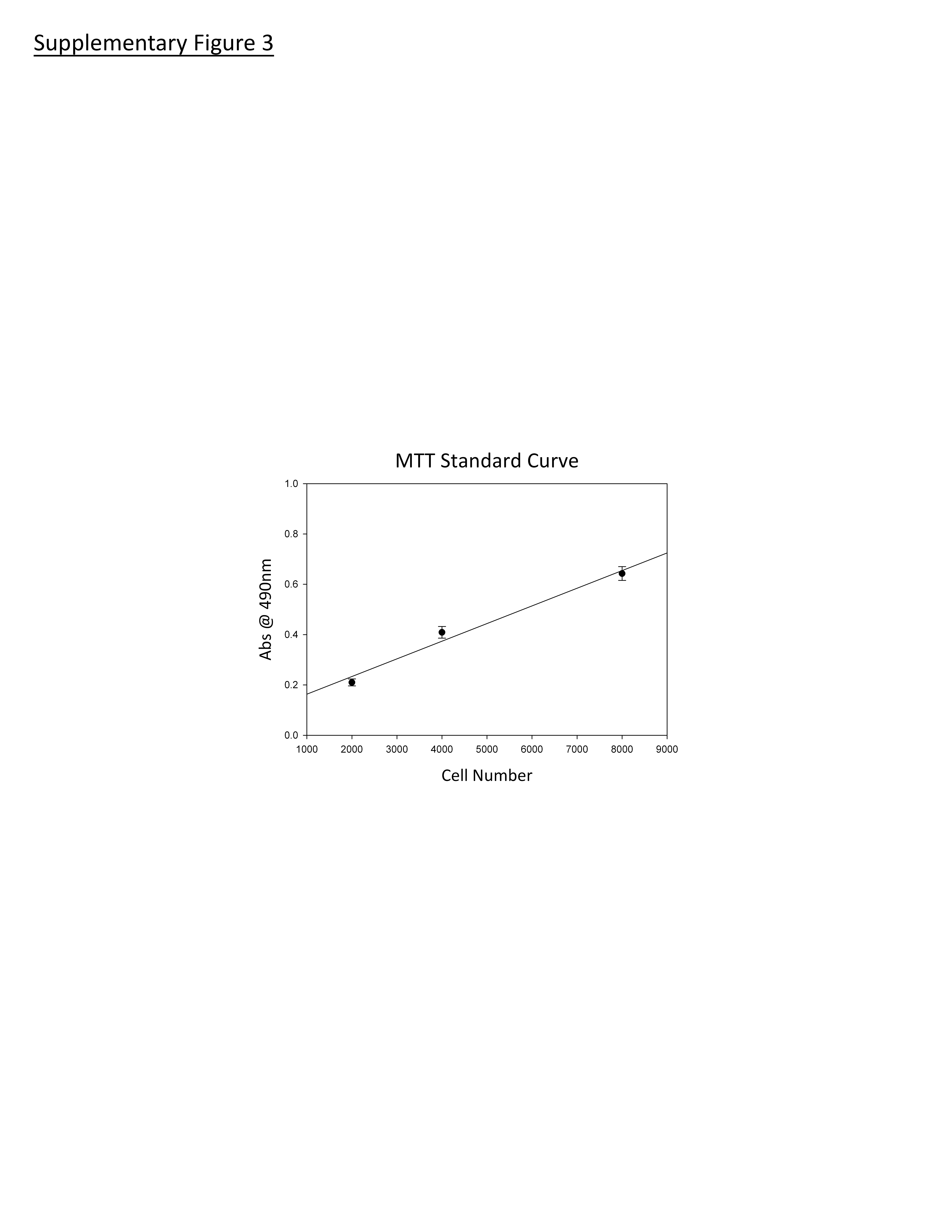

Supplement: Supplementary file 3 — Additional file 3: Figure S3. Standard curve generated from the averaged absorbance readings at 490 nm of two-thousand, four thousand and eight thousand 661W cells. Points represent average absorbance ± SEM at each cell density (n = 20). [file 12868_2017_346_MOESM3_ESM.png]
